# The ABCDE (Avoid Shaming/Personal Opinions, Build a Rapport, Choose a Communication Approach, Develop a Debriefing Content, Ensure the Ergonomics of Debriefing) Approach: A Simplified Model for Debriefing During Simulation in Emergency Medicine

**DOI:** 10.7759/cureus.34569

**Published:** 2023-02-02

**Authors:** Gunaseelan Rajendran, Sasikumar Mahalingam, Aswin K, Ezhilkugan G, Nithya B, S. Manu Ayyan, Balamurugan Nathan

**Affiliations:** 1 Emergency Medicine, Aarupadai Veedu Medical College and Hospital, Puducherry, IND; 2 Emergency Medicine, Jawaharlal Institute of Postgraduate Medical Education and Research, Puducherry, IND

**Keywords:** content for debriefing, educational factors in debriefing, human factors in debriefing, emergency medicine, medical education, patient simulation, debriefing approach, abcde approach

## Abstract

Several debriefing models have been described in the literature. However, all these debriefing models are designed in the general medical education format. Hence, for people involved in patient care and clinical teaching, sometimes it may become tedious and difficult to incorporate these models. In the following article, we describe a simplified model for debriefing using the well-known mnemonic ABCDE. The ABCDE approach is expanded as follows: A - Avoid Shaming/Personal Opinions, B - Build a Rapport, C - Choose a Communication Approach, D - Develop a Debriefing Content, and E - Ensure the Ergonomics of Debriefing. The unique thing about this model is that it provides a debriefing approach as a whole rather than only the delivery. It deals with human factors, educational factors, and ergonomics of debriefing, unlike other debriefing models. This approach can be used for debriefing by simulation educators in the field of emergency medicine and also by educators in other specialties.

## Introduction and background

Simulation in healthcare is gaining much importance, especially in the field of medical education. Simulation as a tool for medical education plays a vital role in emergency medicine [1]. The curriculum for emergency medicine offers much scope for simulation as a medical education tool, as the traditional teaching methodologies of bedside teaching are often impractical and unethical when attending to critically ill patients [2-6]. One of the key areas of simulation is debriefing. Various human and psychological factors are associated with debriefing after simulation. More often than not, the debriefing becomes a fault-finding mission rather than a proper debriefing session. Hence, to streamline the debriefing process, especially for emergency physicians, we propose a debriefing approach in the format of ABCDE (Avoid Shaming/Personal Opinions, Build a Rapport, Choose a Communication Approach, Develop a Debriefing Content, Ensure the Ergonomics of Debriefing), which is traditionally known to emergency physicians.

## Review

The ABCDE approach

Our ABCDE approach includes A - Avoid Shaming/Personal Opinions, B - Build a Rapport, C - Choose a Communication Approach, D - Develop a Debriefing Content, and E - Ensure the Ergonomics of Debriefing. The simplified approach deals with four main factors involved in debriefing: 1. human factors, 2. educational factors, 3. communication factors, and 4. ergonomics. In our ABCDE approach, the first two letters (A and B) deal with human factors. The letter C deals with communication factors; the letter D deals with educational factors, and the letter E deals with ergonomics (Table 1).

**Table 1:** The “ABCDE” approach to debriefing

ABCDE approach	
A - Avoid Shaming/Personal Opinions	1. Debriefing with good judgment
B - Build a Rapport	Establish psychological safety: 1. Face sensitivities. 2. Sociality rights and obligations. 3. Interactional goals. 4. Clarify expectations. 5. Maintain confidentiality
C - Choose a Communication Approach	1. Directive feedback approach. 2. Plus/delta approach. 3. After Action Review (AAR) approach. 4. Advocacy-inquiry-focused facilitation
D - Develop a Debriefing Content	1. The how and what questions. 2. The crisis resource management model
E - Ensure the Ergonomics of Debriefing	1. Dedicated debriefing room. 2. Verbal vs. video-assisted debriefing. 3. Uniprofessional vs. multiprofessional team

A - Avoid Shaming/Personal Opinions

The facilitators use the interval between the brief (the actual simulation) and the debrief to analyze the brief critically. After analyzing the brief, the facilitators may use three different debriefing styles: 1. judgmental debriefing, 2. non-judgmental debriefing, and 3. debriefing with good judgment. The judgmental type of debriefing should not be used during a simulation. This style of debriefing is considered offensive as it directly accuses the learner. The learner is cornered and pushed into a defensive mechanism that creates a lifelong emotional and psychological impact. In the judgmental type of debriefing, the facilitator has an active role, speaks with authority, and has accusative highlighters like “You” and “You failed to notice.” Avoid the judgmental style of debriefing at any cost in debriefing [7,8]. 

The non-judgmental style of debriefing is more of an open-ended question in which the facilitator helps the learner analyze his critical actions. Non-judgmental debriefing has the advantage that it avoids directly blaming and hurting the learner. But the serious drawback of a non-judgmental debriefing is the failure to communicate crucial learning points to the learner at the end of the brief. Instead, it leaves the learner with more questions than answers. It confuses the learner as to whether what he has done during the brief is right or wrong. It also does not analyze the trainee’s thought process when performing critical actions during the simulation [7,8].

Hence, the better style of delivering the debrief is to debrief with good judgment [9]. A debriefing with a good judgment style incorporates an advocacy-inquiry style to elicit the trainee’s thought process at the time of the critical action being done [8]. Here, the instructors do not assume that their personal opinions are a single truth; rather, it challenges the instructors’ and trainees’ opinions to reach a consensus. This style is neither facilitator-centric nor learner-centric. It gives equal space and importance to both individuals’ opinions. Using video-assisted debriefing helps the facilitator deliver the debrief with good judgment.

B - Build a Rapport

The literal meaning of the word rapport is "a friendly and harmonious relationship". Building a rapport between the facilitator and trainee is crucial during debriefing. It creates trust between the facilitator and the trainee, helping the facilitator and learner interact without a sense of threat or shame.

According to the rapport model developed by Spencer-Oatey, there are three integral components to rapport management [10]. These include 1. Face sensitivities. 2. Sociality rights and obligations. 3. Interactional goals. The face is a big part of one’s identity. It is concerned with the desire for autonomy (negative face) and the desire for approval (positive face). Face sensitivities are associated with respect, honor, status, reputation, and competence. One feels threatened when their face values are challenged for positive attributes and acknowledged for negative attributes [11 -13]. The learner feels respected when the face values are respected [14]. Face values are disturbed when feedback is given disrespectfully to the learner or when the learner is forced into uncomfortable scenarios [11]. Hence, one should try avoiding these kinds of behaviors during debriefing.

Sociality rights and obligations are concerned with roles, norms, behavioral conventions, and protocols [11]. It concerns the facilitator’s and learner’s expectations about being treated equally and fairly. It is also concerned about respecting the fictional contract made during the pre-brief. When these social rights and obligations are not met, it creates a sense that the facilitator’s main motive is to degrade and damage the learner’s self-worth. Sociality rights are disturbed when the facilitator imposes personal opinions rather than acknowledging the learner’s thought process and forcing inexperienced learners to contribute the same as experienced learners [15]. 

Interactional goals refer to what the learner desires to achieve during the debriefing session (the learners’ goal during the interaction or simulation). This can be achieved by clearly stating the debriefing session’s objectives or asking the learners about the debriefing process’s agenda and prioritizing those agendas. Interactional goals can be easily achieved by designing a learner-centered simulation and debriefing. A learner-centered debriefing can easily achieve all three elements of rapport building and management. 

C - Choose a Communication Approach

Various communication strategies can be employed during debriefing. Four communication approaches are popular as a dominant educational performance review. These include 1. directive feedback approach, 2. plus/delta approach, 3. After Action Review (AAR) approach, and 4. advocacy-inquiry-focused facilitation. 

I. Directive feedback: feedback and debriefing are at opposite ends of the spectrum. Feedback is unidirectional, whereas debriefing is bidirectional [16]. Feedback is facilitator-centered, wherein the facilitator provides information to close the performance gap observed during an activity. Feedback is not a debriefing approach. But feedback can be used as a communication approach when there is a shortage of time (during OSCE) or when the learning objective does not require analysis of frames performed by the learners. For example, during RSI, the choice of induction agents becomes evident and didactic, like ketamine for asthmatics. Hence this form of communication is less interactive, passive, and facilitator-based [17]. The advantage of directive feedback as a communication approach is that it is rapid and close to the performance gaps of didactic scenarios. The disadvantage of directive feedback as a communication approach is that the learner’s intention behind the actions is completely ignored. It is a one-way communication process, and there is no discussion [18].

II. Plus/delta: the plus/delta approach is a communication approach where the plus comprises the positivities (what did work in this scenario?) of the simulation scenario, and the delta represents the negativities (what could have been performed better?) of the simulation [19]. The plus/delta approach identifies and closes the performance gaps during the simulation. It is a learner-centered communication approach [18]. The plus/delta approach is a fast and easy way to communicate. The plus/delta approach gives many solutions in a short period. But the facilitator and the learner can get lost as too many suggestions may be given, which may take the debriefing to some other area and not the intended learning objectives. The plus/delta also does not allow the learner to self-react as the facilitator closes the gap most of the time.

III. After Action Review (AAR): AAR is an extension of the plus/delta communication approach wherein, along with the closure of the performance gap, the intentions or the thought process behind the actions are also explored [20,21]. In this form, the action is reviewed by two questions: what is ideal? And what has happened? This allows the learners to understand the difference and the performance gap. To close the performance gap, the facilitator can ask questions like, why was there a difference? and what can we learn from this? [18]. The advantages of AAR are that (i) it is learner-driven, (ii) it involves interactive discussion, and iii) it analyzes the intention behind the actions.

IV. Advocacy-inquiry-focused facilitation: the advocacy-Inquiry-focused communication approach is similar to debriefing with good judgment [8,9]. It is also learner-driven, and it also explores the intention behind the actions during the simulation. The communication between the facilitator and the learner occurs with advocacy (I observed this, and I’m concerned about this) followed by an inquiry (I am curious to know why) into the advocacy, thereby analyzing the action’s intentions. This allows the facilitator and learner to close the gaps in the observed performance during a simulation. 

D - Develop a Debriefing Content

The debriefing content deals with the “How, What, When, and Where” of the debriefing session. Good content always guarantees success. Similarly, if the content is good during debriefing, the simulation’s desired outcome can be achieved. Good content has some inherent qualities. These qualities include a clear and concise message that is evidence-based. To build good content, the debriefer should answer specific basic questions. These include “How, What, Who, When, and Where.”

The How question mainly deals with the human factors of the simulation. The How questions for developing the debriefing content are as follows: how did the simulation end? Chaotic or Good? How did the team leader perform? How did the team perform? How effective was the communication between the team? How effective was the team in carrying out the given roles? How was the overall attitude of the team towards each other? How was the interpersonal respect between the team members? How effective was the team in adapting to the hurdles in the scenario? [22]

The What question mainly deals with the educational factors and academics of the sim. The “what about” questions for developing the debriefing content are as follows: what are the predefined objectives of the simulation session? What are all the predefined objectives that were met? In what ways did the learner meet each objective? What are the alternative ways of meeting those predefined objectives? What were the thought processes that led to the observed action? What are the positive actions? What are the negative actions? What can be changed? Attitude, knowledge, and interpersonal communication? [22].

The Who, When, and Where questions mainly deal with the ergonomics of the debriefing. The Who, When, and Where questions for developing the ergonomics of debriefing are as follows: who is going to debrief? (Single facilitator or multiple facilitators for each group of learners, i.e., learners of nursing cadres will be debriefed by a nursing facilitator, and learners of paramedical cadres will be debriefed by a paramedic facilitator). Who is receiving the feedback? (Whether the learners who participated in the simulation or all the learners who were present during the simulation). When is the debriefing intended to take place? (At the end or at the end of each critical action). When can the facilitator interrupt? (At the end or at the end of each critical action, LIVE DIE REPEAT model) [23]. Where is the debriefing intended to take place? (In situ or in a designated area for debriefing or simulation center)

Overall, the content of the debriefing mainly depends on the predefined learning objective. When the facilitator feels that certain predefined objectives are met with exceptional activities and certain predefined objectives are not met, he can then inquire into the learner’s frames for each activity. He can then reinforce the frames that led to positive actions with positive comments and change the frames, leading to negative actions by asking the learner to analyze the frames and didactics. 

E - Ensure the Ergonomics of Debriefing

Ergonomics plays a vital role in debriefing. It is ideal to have a dedicated room for debriefing. This room should be well-lit. It should contain a table and chairs for all the learners and the facilitator. It should have a whiteboard attached to the wall. It should also contain a wall-mounted TV to facilitate video-based debriefing. In many centers, a dedicated room may not be available. In such a scenario, debriefing can take place in the room where the simulation was carried out. But the mannequins and simulation equipment should be moved, and adequate seating arrangements should be made.

The next important aspect of ergonomics in debriefing is the debriefing of a uniprofessional and multi-professional team. A multi-professional team needs multi-professional debriefers. Multi-professional debriefings are challenging, even for experienced debriefers. Emergency medicine simulations always involve a multi-professional team in order to make them high-fidelity simulations. Hence, while debriefing, it is important to keep certain basic principles in mind. The debriefer should anticipate various logistical problems, and hence he/she should balance diversity by giving equal opportunities to all the learners. This should be achieved by giving equal importance and opportunities while developing the simulation scenarios. When addressing the performance gap in a particular area, importance can be given to the expert in that field. 

The next ergonomic challenge in debriefing is making use of audio-visuals. Video-assisted debriefing is an emerging idea. Like any other innovation, video-assisted debriefing has its advantages and disadvantages. A meta-analysis by Zhang et al. on video-assisted debriefing concluded that video-assisted debriefing improves the learner’s experience, attitude, and performance but does not significantly affect their knowledge acquisition compared to verbal debriefing [24]. Table 2 provides a comparison between PEARLS (Promoting Excellence And Reflective Learning in Simulation) debriefing and the ABCDE approach of debriefing.

**Table 2:** Comparison between PEARLS debriefing and ABCDE approach of debriefing

	PEARLS debriefing	ABCDE approach
Setting the scene	Done	Done in ‘A & B’: Avoid shaming and Build a Rapport
Reactions	Done	Done by using directive feedback in the communication approach in the ‘C’ part
Description	Done	Done by using Plus/delta or advocacy Inquiry in the ‘C’ part
Analysis	Done	Done in D part
Application/summary	Done	Not done

## Conclusions

The ABCDE mnemonic is a tried and tested mnemonic for an emergency physician. This mnemonic is the bread and butter for the emergency physician. Hence, incorporating this mnemonic and developing an approach to debriefing can be very useful for emergency physicians involved in debriefing. There are many debriefing models with mnemonics. We have provided a simplified debriefing model with the time-tested ABCDE mnemonic.

In the ABCDE approach for debriefing that we have developed, we focus on the content of debriefing and provide a comprehensive approach to debriefing with an emphasis on human and educational factors. Our approach can be used for debriefing by simulation educators in the field of emergency medicine and educators in other specialties.
